# Reducing Endogenous Labile Zn May Help to Reduce Smooth Muscle Cell Injury around Vascular Stents

**DOI:** 10.3390/ijms23095139

**Published:** 2022-05-05

**Authors:** Zheng Zeng, Yinhong Xie, Li Li, Huanran Wang, Jianying Tan, Xia Li, Qihao Bian, Yu Zhang, Tao Liu, Yajun Weng, Junying Chen

**Affiliations:** 1 Key Laboratory of Advanced Technology of Materials, Ministry of Education, School of Materials Science and Engineering, Southwest Jiaotong University, Chengdu 610031, China; zhengzeng@my.swjtu.edu.cn (Z.Z.); yinhongxie@my.swjtu.edu.cn (Y.X.); lili1993@my.swjtu.edu.cn (L.L.); huanran@my.swjtu.edu.cn (H.W.); jianying1128@126.com (J.T.); 17361018818@163.com (X.L.); qhbian0310@163.com (Q.B.); yuzhang180@163.com (Y.Z.); 2 Medical College of Acu-Moxi and Rehabilitation, Guanzhou University of Chinese Medicine, Guangzhou 510006, China; lt045021@gzucm.edu.cn

**Keywords:** endogenous zinc, necrosis, cell injury, vascular stent, Zinquin

## Abstract

Vascular stent service involves complex service environments and performance requirements, among which the histocompatibility of the stent could seriously affect the therapeutic effect. In the pathology of vascular disease, the thin fiber cap is easily ruptured, exposing the necrotic core below, and triggering a series of dangerous biochemical reactions. In contrast, the thin neointima, considered an essential structure growing on the stent, may evolve into vulnerable plaque structures due to lesions induced by the stent. Therefore, the reduction of necrosis around the stent below the thin neointima is indispensable. In this work, different cell model experiments suggested that the content of endogenous labile Zn positively correlated with cell injury. Zinquin-Zn fluorescence experiments and zinc ion channels research suggested that the change in the content of endogenous labile Zn in smooth muscle cells is affected by different stent coatings. The content of endogenous labile Zn in cells negatively correlated with cell viability. Animal experiments indirectly verified the increase in endogenous labile Zn by detecting the expression of Zn regulatory protein (metallothionein) in the necrotic tissues. Reducing the content of endogenous labile Zn may favor a reduction in smooth muscle cell injury and necrosis. This biochemical mechanism is effective in improving the therapeutic effect of vascular stents.

## 1. Introduction

The structure of human society has shown an aging trend. Moreover, vascular diseases have gradually started affecting the elderly at younger ages [1]. Although cardiovascular stents have been developed for decades [2] as a necessary treatment for cardio-blood diseases, there are still many problems. At present, the primary design strategy for the functional design of the current stent is antithrombosis and re-endothelialization. However, the latest data shows that its effect regarding the prevention of cardiovascular disease is not very satisfactory [3]. It is well known that intact endothelialization is conducive to blood flow, but endothelial cells (ECs) grow on the smooth muscle layer. Inhibition of the growth of smooth muscle cells (SMCs) is a common strategy [4] for preventing in-stent restenosis while the strategy may also damage SMCs and then affect ECs’ growth. This problem can be traced to the second-generation vascular stents [5].

In stent design, it may still be necessary to seek other theories. Pathologically, a large proportion of cardiovascular disease originates from atherosclerotic plaques, which have a necrotic core [6,7]. The thinner the fiber cap on the necrotic core, the more vulnerable the plaque, and the more easily it ruptures, exposing the necrotic core in the plaque and forming a thrombus [8]. In contrast, the thin neointima, considered an essential structure growing on the stent, may evolve into vulnerable plaque structures due to lesions induced by the stent. Therefore, accurate biochemical mechanisms for detecting the biocompatibility of stents are required.

As an essential trace element for life, endogenous Zn is associated with the survival, growth, and regulation of protein functions [9], thus impacting on the overall metabolic efficiency. Slight changes in the zinc ions in Zn-containing endogenous substances can interfere with its functions [10]. The zinc ions in cells are very sensitive to the outside world and can change significantly within a few minutes. In PD Zalewski’s works, the influx of exogenous Zn into lymphocyte cells inhibited DNA fragmentation induced by colchicine. In contrast, the content of endogenous labile (free or thermodynamically exchangeable) zinc ions in HL60 cells positively correlated with DNA fragmentation [11]. The same increase in intracellular zinc ions has the opposite effect on DNA fragmentation. This may be due to the different sources (exogenous or endogenous) of zinc ions, such as the influx of exogenous Zn to inhibit Ca^2+^/Mg^2+^ nuclease activity to suppress DNA fragmentation in human chronic lymphocytic leukemia cells [12]. Another source, such as the release of endogenous Zn to facilitate DNA fragmentation and the release of endogenous Zn originating from cytokines, enzymes, or the cellular structure, disturb their functions to cause an apoptotic cascade [12,13,14]. In addition, the changes in cellular Zn channels are very sensitive to the external environment [13]. Therefore, from the perspective of cell biochemistry, it is worth considering whether the cytocompatibility can be accurately judged in advance through the change in endogenous Zn.

**Figure 1:**
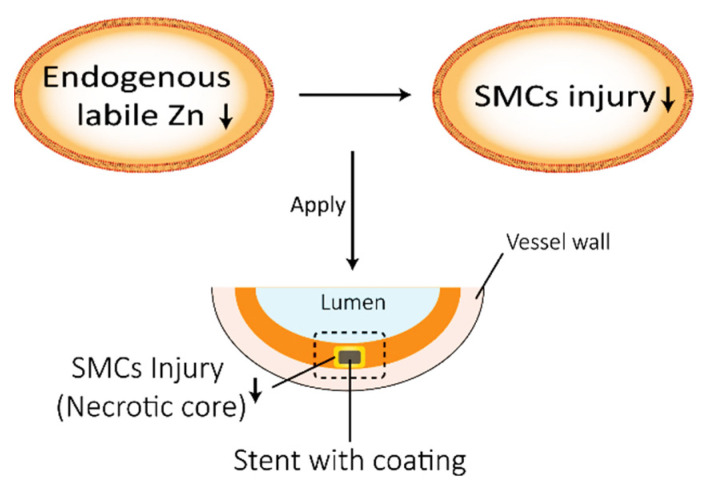
Schematic illustration showing the potential relationship between endogenous labile Zn and cell injury and its potential application in stents (SMCs, smooth muscle cells).

In this work, the relationship between endogenous labile zinc and cell viability was firstly investigated using endogenous labile zinc ions in different cell models. Secondly, the relationship between endogenous labile zinc and cell viability was investigated after the cells were cultured on stent coatings with or without exogenous Zn (the stent coating technology is based on previous work [14]). Our goal was to investigate the biochemical relationship between the change in the endogenous zinc content in smooth muscle cells and the cell viability, and to determine whether this biochemical mechanism could improve the therapeutic efficacy of stents by reducing tissue necrosis (Figure 1).

## 2. Results

### 2.1. Cell Injury Model and the Changes in the Endogenous Labile Zn Content

**Figure 2:**
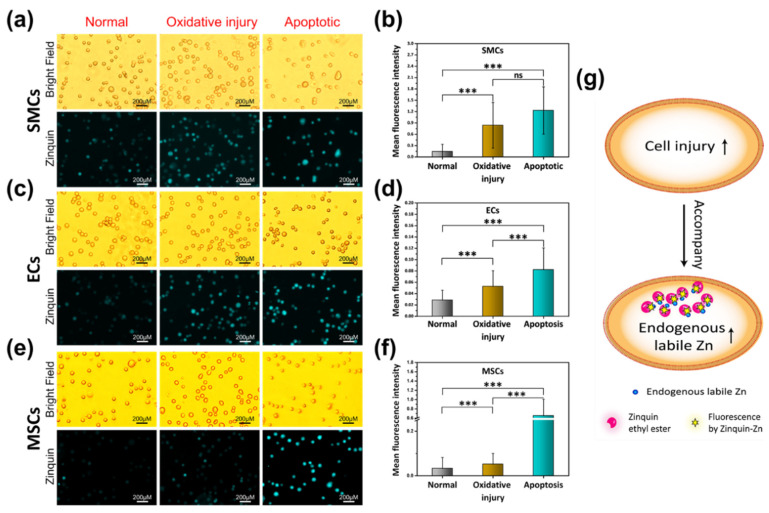
Zinquin-Zn fluorescence and the mean fluorescence intensity (MFI) of Zinquin-Zn in different cell injury models, including a normal group, oxidative injury group, and apoptosis group. Zinquin-Zn fluorescence for SMC (**a**), ECs (**c**) and MSCs (**e**) in different cell models. The MFI of Zinquin-Zn for SMC (**b**), ECs (**d**) and MSCs (**f**). (**g**) Cell injury accompanied by an increase in endogenous labile Zn (SMCs, smooth muscle cells; ECs, endothelial cells; MSCs, mesenchymal stem cells; data were analyzed using 1-way ANOVA, *** *p* < 0.001; ns, not significant).

To explore the endogenous labile Zn content in different cell models, the probe reagent Zinquin-ester was utilized. Zinquin-ester was used as a probe for the labile Zn in living cells through the fluorescence of Zinquin-Zn. The ester group can be hydrolyzed by living cells to ensure intracellular retention of Zinquin [15]. Firstly, the fluorescence of the probe reagent Zinquin [16] to different metal ions, including Zn^2+^, Mg^2+^, Fe^2+^, Fe^3+^, Cu^2+^, Ca^2+^, and Al^3+^, was tested (Figure S1). An excitation wavelength of 333 nm was used to record the emission spectra from 350–550 nm. Zinquin-Zn showed an almost 9-fold increase in the fluorescence intensity, with λ_MAX_ centered at 424 nm compared to the other metal ions. To study the relationship between the change in the content of endogenous labile zinc ions and the cell viability state, different cells, including SMCs, ECs, and mesenchymal stem cells (MSCs), were digested and treated by different injury models. As a traditional cell culture method in biology, cells cultured on a tissue culture plate (TCP) can help us to understand the normal indicators (e.g., Zinquin-Zn, cell viability) of cells. As well-known among all three cell models, the best cell viability value was the normal group, followed by the oxidative injury group, and the worst cell viability was observed in the apoptotic group. As the Zinquin-Zn fluorescence images and the semi-quantitative fluorescence intensity statistics results (determined by image-Pro plus software) show in Figure 2a,b (SMCs), Figure 2c,d (ECs), and Figure 2e,f (MSCs), the highest mean fluorescence intensity (MFI) was observed in the apoptosis group compared to the other conditions of the cell model groups. Both the apoptosis group and the oxidative injury group showed intense fluorescence compared to the normal group. Overall, these results demonstrate the phenomenon of increased endogenous labile Zn in response to cell injury (Figure 2g).

### 2.2. Preparation of the Coatings Containing Exogenous Zn 

**Figure 3:**
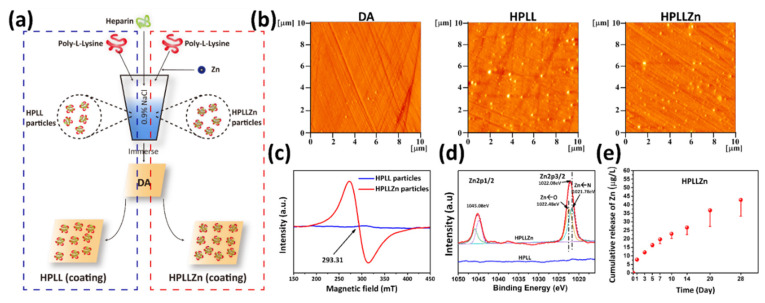
(**a**) Schematic illustration of the preparation of HPLL and HPLLZn. (**b**) AFM images of DA, HPLL, and HPLLZn. (**c**) The EPR spectra of HPLLZn particles used for coating preparation. (**d**) Zn 2p XPS spectrum of coatings. (**e**) Quantitative characterization of Zn leaching from HPLLZn (DA, dopamine coating; HPLL, heparin/poly-L-lysine coating; HPLLZn, heparin/poly-L-lysine/Zn coating).

To directly demonstrate the potential relationship between the endogenous labile Zn content and the cell viability on the stent coating, based on previous work [14,17], exogenous Zn was introduced in the stent coating to observe its effect on endogenous Zn in the cells. The introduction of exogenous zinc ions into the coating reversely proved the changes in the cell’s endogenous labile Zn. The technological process of the introduction of Zn ions into the anticoagulation coating is illustrated in Figure 3a. Dopamine coatings (DA) were immersed in heparin/poly-L-lysine (HPLL, Zn-free) particles and heparin/poly-L-lysine/Zinc (HPLLZn, Zn-containing) particles to prepare the two coatings separately. The maximum Zn ion concentration was optimized to ensure the cell compatibility of SMCs and ECs (Figure S2). The anticoagulant properties of the optimized coatings were tested in advance (Figure S3) to further conduct an in vivo experiment. The atomic force microscope (AFM) and quartz crystal microbalance with dissipation (QCM-D) (Figure S4) results show that HPLL particles and HPLLZn particles were successfully immobilized on the DA coating (Figure 3b) and the grafting amount of HPLLZn particles was less than that of HPLL particles. No signals were observed in the complexes without Zn while a clear electron paramagnetic resonance (EPR) signal was observed in complexes with Zn at 293.31 mT with a g value of 2.4003, suggesting a chelating reaction occurred in the particles (Figure 3c). XPS analysis was used to determine the elemental composition of the coatings. As shown in Figure S5, a new peak binding energy of Zn at 1022.08 eV was observed in the full spectrum after immobilization of the HPLLZn particles. Figure 3d shows the further curve fitting of the high-resolution spectrum of Zn2p, which suggests that Zn existed in the Zn-N state at a binding energy of 1022.48 eV and in the Zn-O state at a binding energy of 1021.78 eV [18]. Regardless of the state, zinc ions were successfully introduced into the HPLLZn coating. The ICP-MS result shows that the Zn ions in HPLLZn were released at a rate ranging from 0.40 to 4.11 ng·cm^−2^·day^−1^ (Figure 3e). Thus, HPLL without zinc ions and HPLLZn, which can release Zn into the environment, were successfully prepared.

### 2.3. Changes in Endogenous Labile Zn in SMCs and Cytocompatibility Evaluation

**Figure 4:**
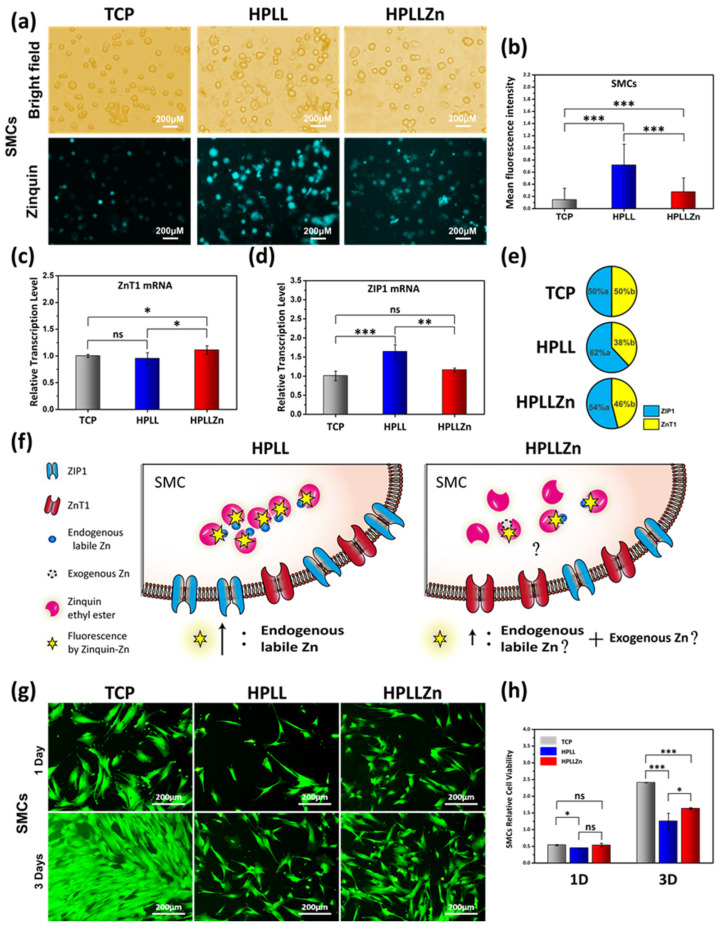
(**a**) Zinquin-Zn fluorescence in smooth muscle cells after contact with different coatings. (**b**) Semi-quantitative statistics of the mean fluorescence intensity (MFI) of Zinquin. The relative transcription level of mRNA is related to the Zn ion channel, (**c**) *ZnT1* for efflux of the Zn ion, and (**d**) *ZIP1* mRNA for the influx. (**e**) The average relative ratio of the zinc channel mRNA transcription levels. (**f**) Schematics of the sources of the fluorescent. (**g**) Rhodamine 123 fluorescence images of SMCs’ growth and (**h**) the cell viability after incubation for 1 day and 3 days (TCP, tissue culture plate; data were analyzed using 1-way ANOVA, * *p* < 0.05, ** *p* < 0.01, *** *p* < 0.001; ns, not significant).

As the stent will eventually be encapsulated by fibrous tissue [19], we aimed to investigate the smooth muscle cells (SMCs) on the coatings. In the experiment of smooth muscle cells’ (SMCs’) contact with different coatings, except for HPLLZn, no exogenous Zn was present in the environmental system of HPLL and TCP. Compared with the most minor Zinquin-Zn fluorescence in SMCs on TCP, significantly increased Zinquin-Zn fluorescence was observed in SMCs on HPLL and HPLLZn, and the fluorescence increased the most on HPLL (Figure 4a,b). To further investigate the changes in intracellular Zn, the *ZnT1* transporter, which effluxes intracellular Zn, and *ZIP1* transporter, which imports Zn [20], were measured. Compared with TCP, the HPLLZn coating significantly increased the *ZnT1* mRNA transcription level while the HPLL coating intensely increased the *ZIP1* mRNA transcription level (Figure 4c,d). The average cellular ratio of *ZnT1* mRNA to *ZIP1* mRNA was deduced according to Equation (1), indicating that SMCs on HPLL and HPLLZn tended to promote an influx of Zn compared to TCP, and HPLL facilitated this influx the most (Figure 4e). The changes in the Zn ion channels corroborate the changes in the intracellular Zn and the degree of Zn influx tendency is positively correlated with the endogenous labile Zn content of the cell. This may be due to an adjustment in the feedback of SMCs to the loss of Zn in the endogenous substances [21].

In this experiment, TCP was considered as the negative control group for the endogenous labile Zn research. Compared with normal SMCs, which showed almost no Zinquin fluorescence on TCP, the enhancement in the Zinquin fluorescence in SMCs on HPLL was completely caused by an increase in endogenous labile Zn. Although this work cannot determine the precise source of Zn in the fluorescence of HPLLZn, the source of this part of Zn is at most the sum of endogenous labile Zn and exogenous Zn. Because the Zinquin fluorescence intensity in HPLLZn is less than HPLL, it can be judged that HPLLZn cells have less endogenous labile Zn than HPLL (Figure 4f). 

Rhodamine 123 staining (Figure 4g) and cell counting kit-8 (CCK-8) (Figure 4h) measurement were used to investigate SMCs’ viability on the coatings. On the first day, the total viability of SMCs on TCP was not significantly different from that on HPLLZn, but it was significantly increased compared to that on HPLL. No significant difference was observed between HPLL and HPLLZn. On the third day, the cell viability on TCP was significantly increased compared to HPLLZn and HPLL, and the gap gradually widened. The cell viability on HPLLZn was also significantly enhanced compared with HPLL. Combined with the endogenous labile Zn fluorescence results in Figure 4b, the content of cellular endogenous labile zinc ion is negatively correlated with cell viability.

### 2.4. Reactions in the Animal Body and the Application in Vascular Stent

**Figure 5:**
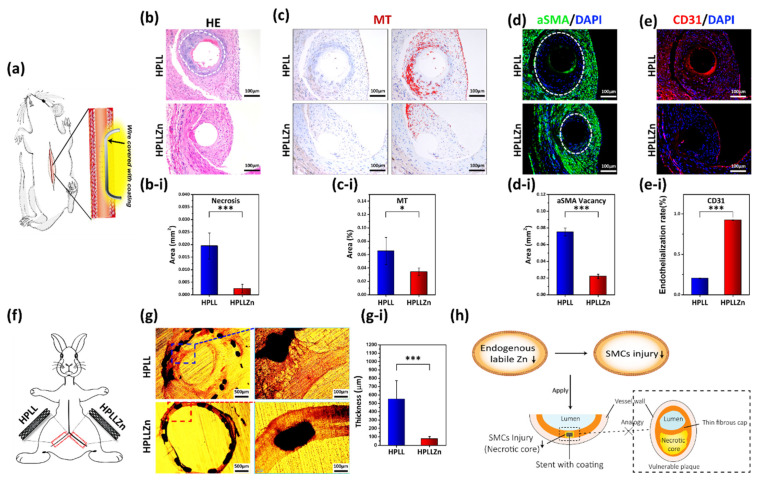
(**a**) Schematics of stainless-steel wires with different coatings implanted into Sprague Dawley rats’ abdominal aorta. (**b**) Hematoxylin and eosin staining and (**b-i**) quantification of the necrotic area surrounding the different coatings (the white dotted line shows the necrotic area). (**c**) Immunohistochemical of metallothionein protein expression and (**c-i**) semiquantitative analysis of the area (red marks regions with high MT expression). (**d**) The expression edge of α-SMA (green) and (**d-i**) quantification of the area without αSMA in the tissue close to the coating after a 1-month implantation (the white dotted line shows the edge closest to the coating). (**e**) Immunofluorescence staining of CD31 and (**e-i**) the endothelialization integrity. (**f**) Schematics of vascular stents with different coatings implanted in New Zealand white rabbits’ iliac artery. (**g**) Histomorphometric analysis of stents after a 1-month implantation and (**g-i**) quantification of the mean neointimal thickness. (**h**) The potential mechanism of this rule applies to the cardiovascular stent. (HE, hematoxylin and eosin stain; MT, metallothionein; α-SMA, α-smooth muscle actin; CD31, cluster of differentiation 31; DAPI, 2-(4-Amidinophenyl)-6-indolecarbamidine dihydrochloride; data were analyzed using 1-way ANOVA, * *p* < 0.05, *** *p* < 0.001; ns, not significant).

To observe the actual effect in animals, 316L stainless-steel wires covered with different coatings were implanted into the abdominal aortas of *Sprague Dawley rats* (Figure 5a). Hematoxylin-eosin staining showed pathological necrosis (without healthy parenchymal cells [22]) in the tissue around HPLL while healthy tissue grew around HPLLZn (Figure 5b). The average area of necrosis was 0.019 ± 0.005 mm^2^ with HPLL and 0.002 ± 0.002 mm^2^ with HPLLZn. The main functions of metallothionein (MT) are to regulate the Zn metabolism and balance and the storage of metal ions to protect against heavy metal injury in the human body [23,24]. When excessive Zn is present in cells, MT participates in the balance and storage of zinc ions in the human body, and biosynthesis is inducible [25]. As the ester group could not be hydrolyzed by necrotic tissue and no complete cell membrane was observed to ensure intracellular retention of Zinquin, the MT protein observed here could indirectly give clues regarding the change in the labile zinc content in cells. The enhanced expression of MT in HPLL indicated that metal balancing, and excess endogenous labile Zn have occurred (Figure 5c). For HPLLZn, minor MT immunohistochemical staining was observed. Percentage quantification of the MT expression area in the new tissue (Figure 5c-i) showed that the MT expression around HPLL was 0.07 ± 0.02%, which is significantly enhanced when compared to 0.03 ± 0.01% of HPLLZn. The α-SMA protein (green) suggests the contraction phenotype of smooth muscle cells [26]. Figure 5d shows that the edge of the α-SMA expression is closer to HPLLZn, suggesting a better biological affinity for SMCs than HPLL. The area in which no SMCs are located around HPLL is 0.08 ± 0.01 mm^2^ and HPLLZn is 0.02 ± 0.01 mm^2^ (Figure 5d-i). Endothelialization is a routine evaluation index used to assess the service effect of vascular stents. CD31 fluorescent staining of endothelial cells showed that the endothelialization rate of HPLLZn was 0.92 ± 0.01%, which is significantly higher than the 0.2 ± 0.01% of HPLL (Figure 5e,e-i).

We covered the coatings with cardiovascular stents, and then stents were implanted in the iliac arteries of *New Zealand white rabbits* under a high-fat diet (Figure 5f). Cross-sections of the blood vessels were obtained after implantation for 30 days. As the result shows, a severe tissue lesion and hyperplasia developed in HPLL while for HPLLZn, new tissue was dense and showed a close fit (Figure 5g). The quantification result shows that the thickness of the neointima in HPLL was 551.46 ± 220.37 μm, which is significantly thicker than HPLLZn at 74.96 ± 27.57 μm (Figure 5g-i). The schematic describes the potential biochemical mechanism of the stent coating. Through controlling the endogenous labile Zn, the necrotic tissue around the stent was reduced to avoid the evolution of unstable plaques (Figure 5h).

## 3. Discussion and Conclusions

In this work, we aimed to explore the biochemical mechanism between endogenous labile Zn and SMC viability. This mechanism could be applied to improve the therapeutic effect of vascular stents. 

Firstly, using the specific Zn staining probe Zinquin-ester, it was found that endogenous labile Zn was increased accompanied by cell injury in the three cell models. This cytochemical phenomenon has a certain universality. 

Secondly, the Zinquin-Zn fluorescence results show that the HPLLZn coating containing exogenous Zn did not lead to an increase in the intracellular labile Zn content in SMCs compared to HPLL. For HPLLZn, the source of intracellular labile Zn is, at most, the sum of endogenous labile Zn and exogenous Zn while the increased intracellular labile Zn binding with Zinquin on HPLL can only be caused by a change in endogenous Zn (the stability turns into a labile state). The sharp decrease in intracellular labile Zn in HPLLZn helps to prove the reduction in endogenous labile Zn compared to HPLL. The results of the RNA transcription of cellular zinc ion channels showed that SMCs on HPLL tended to take up more Zn compared to HPLLZn. Considering the lack of exogenous Zn in SMCs cultured on HPLL while there is more endogenous labile Zn, the increase in labile Zn may due to the release of Zn from some endogenous substances. The decrease in Zn utilization for some endogenous substances result in the respond of SMCs to regulate its influx. Combining the content of endogenous labile Zn with conventional cell viability testing, the rule of less endogenous labile Zn resulting in better cell viability also applies for stent coatings. 

In the animal experiments, necrosis around HPLL was observed. This is consistent with the viability of SMCs in vitro. In situ tissue necrosis combined with high expression of MT protein HPLL suggested an excess of endogenous labile Zn. The result of less MT expression in cells surrounding HPLLZn is consistent with a decreased endogenous labile zinc content. The surrounding tissue is relatively healthier, which contributes to a good effect in stent application.

In summary, this work provides a potential biochemical relationship between the endogenous labile Zn content and the viability of SMCs. This biochemical mechanism is effective in improving the therapeutic effect of vascular stents. 

## 4. Experimental and Methods 

### 4.1. Materials and Reagents

Dopamine (DA) and poly-lysine (PLL, MW 150–300 kDa) were purchased from Sigma-Aldrich. Heparin sodium (Hep, MW < 8 KDa, potency > 160 Umg^−1^) was purchased from Shanghai Bioscience and Technology Co. ZnCl_2_ reagent was purchased from Chronchem (Chengdu, China). Zinquin ethyl ester was purchased from Maokangbio (Shanghai, China). A Cell Counting Kit-8 (CCK-8) was purchased from Dojindo Laboratories (Japan). Fast Super Eva Green qPCR Master Mix was purchased from US Everbright (Suzhou, China). A Revert Aid First Strand cDNA Synthesis Kit was purchased from Thermo Fisher Scientific (USA). Rabbit monoclonal anti-mouse α-SMA antibody and rabbit monoclonal anti-mouse CD31 antibody were purchased from Servicebio (China). Rabbit polyclonal to Metallothionein was purchased from Abcam (ab192385).

### 4.2. Coating Preparation

DA coating: This coating was prepared by the deposition of 2 mg/mL^−1^ dopamine solution (dissolved in 10 mM Tris buffer, pH = 8.5) at 20 °C for 12 h. After ultrasonic washing with ultrapure water (UP), the first layer was completed. This operation was repeated three times, forming a three-layer DA coating.

The HPLL and HPLLZn coating preparations were based on previous work [14,17]. Firstly, 0.125 mL of poly-l-lysine (1 mg/mL, dissolved in NS) were mixed with 0.125 mL of NS or 0.125 mL of ZnCl_2_ (5 mM, dissolved in NS) under ultrasonic conditions for 5 min. Then, 0.25 mL of heparin solution (10 mg/mL, dissolved in NS) were added to the mixture to form the particles. Subsequently, the DA coating prepared before was immersed in different particle solutions at 20 °C for 12 h. After three ultrasonic washings with UP, the preparation of the HPLL coating and HPLLZn coating was complete. The samples were stored at −20 °C. 

### 4.3. Electron Paramagnetic Resonance (EPR)

Electron paramagnetic resonance (EPR) spectra were obtained using a Bruker A320 with a microwave bridge. The detection parameters were as follows: receiver gain, 1 × 10^5^; modulation amplitude, 2 Gauss; microwave power, 20 mW; modulation frequency, 100 kHz.

### 4.4. X-ray Photoelectron Spectroscopy (XPS)

XPS was performed to detect the surface chemical compositions of the coating using a K-Alpha X-ray photoelectron spectrometer (Thermo Electron, Waltham, MA, USA).

### 4.5. Inductively Coupled Plasma Mass Spectrometry (ICP-MS)

Different coatings immersed in 1.5 mL of PBS were placed in airtight centrifuge tubes and incubated at 37 °C under constant rotation (70 rpm). PBS was collected and replaced after 1, 3, 5, 7, 10, 14, 21, and 28 days. Quantitative characterization of the Zn release (*n* = 3) was conducted using inductively coupled plasma mass spectrometry (Thermo ICP6300).

### 4.6. Specific Fluorescence Detection with Metal Ions

The concentration of metal ions was 5 mM, and the concentration of Zinquin was 5 µM. Different metal ions were mixed with Zinquin to react for 30 min at room temperature and protected from light. Hitachi F-7000 was used for fluorescence spectrophotometer detection.

### 4.7. Zinquin Staining of Smooth Muscle Cells on Coatings and Measurement of the Fluorescence Intensity

Smooth muscle cells (SMCs) were isolated from a human umbilical artery. The details are available from a previous work [14]. SMCs were cultured on different coatings for 7 h in serum-free high-glucose Dulbecco’s Modified Eagle Medium (zinc-free environment). Then, Zinquin was added and reacted for 30 min at room temperature and protected from light. Washing was carried out to remove excess dye and fluorescence images were taken using fluorescence microscopy (Olympus IX51, Tokyo, Japan).

The fluorescence intensity of Zinquin-Zn in cells was calculated by the mean fluorescence intensity (MFI) value of the fluorescence images through the Image-Pro-Plus software (*n* = 4).

### 4.8. RNA Extraction for RT-PCR and Single-Cell Analysis 

SMCs were separately seeded on the coatings (25 × 10^4^ cells per well) and cultured in high-glucose Dulbecco’s Modified Eagle Medium (DMEM) containing 10% FBS at 37 °C under 5% CO_2_ for 36 h. Cells were rinsed with precooled PBS and Trizol reagent (150 mL) added to the cells at −80 °C for 12 h. After that, 30 mL of chloroform were added to the Trizol and shocked for 15 s. Centrifugation was carried out at 4 °C for 15 min, and 75 mL of isopropanol were added to the upper aqueous phase, which was isolated. The mixture was incubated at 25 °C for 10 min and RNA in the precipitation was centrifuged at 10,000× *g* for 10 min. The RNA precipitation was rinsed with 75% alcohol (150 mL), and RNA was obtained on the tube wall by centrifugation at 7500× *g* for 5 min. The collected RNA was diluted with 30 mL of DEPC and RT-PCR followed the reagent instructions (*n* = 4). The primers used for the RT-PCR are given in Table 1.

It was assumed that the expression level of *ZnT1*mRNA in healthy cells is “a” and the expression level of *ZIP1*mRNA is “b”. The average relative ratio of zinc ion channel mRNA transcription levels was calculated using the following equation:

(1) Relative transcription ratio=(a∗ZnT1mRNA):(b∗ZIP1 mRNA)

### 4.9. Cell Viability Analysis

SMCs were cultured on the different coatings at a density of 5 × 10^4^ cells per/cm^2^, and then the Cell Counting Kit-8 (CCK-8) was used to detect cell viability after 24 and 72 h of culture. Cells were fixed with paraformaldehyde (4%), stained with Rhodamine 123, and photographed with a fluorescence microscope.

### 4.10. Cell Injury Model

Smooth muscle cells (SMCs) and endothelial cells (ECs) were isolated from a human umbilical artery. Mesenchymal stem cells (MSCs) were extracted from mouse bone marrow. H_2_O_2_ solution (100 μM) was used to induce oxidative injury in SMCs for 10 min [27]. Cellular apoptosis was generated in a 55 °C water bath for 10 min [28,29]. After centrifugation, injured cells were stained with Zinquin ethyl ester (5 μM) for 30 min. Excess dye was removed by centrifugation, and the cells were observed with a fluorescence microscope. 

### 4.11. Animal Experiments and Histopathological Analysis

All procedures were performed in accordance with the China Council on Animal Care and Southwest Jiaotong University animal use protocol and in accordance with ethical rules for experimental animals. Both healthy male *SD rats* and healthy male *New Zealand white rabbits* were fed at the Experimental Animal Center of Sichuan University and treated according to the guidelines approved by the Institutional Animal Care and Use Committee of Sichuan University. All animals were purchased from Dashuo Experiment Animals Co., Ltd. (Chengdu, China). Their experimental animal production license was authorized by Sichuan Animal Management Committee, the project identification approval code was “SCXK (chuan) 2020-030”, and the date was 6 March 2020.

First, 316L stainless steel wires (ϕ0.15 mm) covered with different coatings were implanted in the abdominal aorta of male *SD rats*. Pentobarbital sodium was used for animal anesthesia. After retention in the body for 30 days without anticoagulation, the blood vessels implanted with wires were collected and fixed with 4% paraformaldehyde overnight. Then, the blood vessels were embedded in paraffin. Next, 5 mm tissue sections were stained after deparaffinization. Necrosis was quantified by measuring the acellular areas of plaques with Image Pro-Plus software [22]. Data were presented as mean ± SD (*n* = 4).

Next, 316L stainless steel stents (ϕ2.75 mm × 18 mm) covered with different coatings were implanted in the iliac artery of male *New Zealand white rabbits* (3 kg). After retention in the body for 30 days with high-fat feeding, the blood vessels implanted with stents were collected and fixed with 4% paraformaldehyde overnight. Then, the blood vessels with stents were embedded in resin. The thickness of the tissue sections was 350 mm. The tissue structure around the stent was observed using a microscope. Data were presented as mean ± SD (*n* = 4).

### 4.12. Immunofluorescent and Immunohistochemical Staining

The experimental operation was in accordance with a previous work [14] and the reagent instructions were followed.

### 4.13. Thickness Statistics

The hyperplasia thickness was obtained by measuring the cross-sectional view of the stent taken by the microscope using IPP software (*n* = 4).

## Figures and Tables

**Table 1:** Primers for the zinc channel transporters.

Primers		Sequence 5′-3′
*ZnT1*	FW	CCCCGCAGACCCAGAAAAC
	RV	GTTGTCCAGCCCTATCTTCTTC
*ZIP1*	FW	ACTACCTGGCTGCCATAGATG
	FV	GCCCTGACTGCTCCTTGTAAG

## Data Availability

Not applicable.

## Supplementary Materials

Supplementary data to this article can be found online at https://www.mdpi.com/article/10.3390/ijms23095139/s1.
