# Supplementary material for: Reducing Endogenous Labile Zn May Help to Reduce Smooth Muscle Cell Injury around Vascular Stents
Source: Int J Mol Sci. 2022 May 5;23(9):5139. doi: 10.3390/ijms23095139 (PMC9101291; doi:10.3390/ijms23095139)
Supplement: Supplementary file 1 [file ijms-23-05139-s001.zip › ijms-1696337-supplementary.pdf]

Supplementary information

Reducing endogenous labile Zn may help to reduce the smooth muscle cell injury around vascular stent

Zheng Zeng [a, 1], Yinhong Xie [a], Li Li [a], Huanran Wang [a], Jianying Tan [a], Xia Li [a], Qihao Bian [a], Yu Zhang [a], Tao Liu [b, *], Yajun Weng [a, *], and Junying Chen [a, *]

[a] Key Laboratory of Advanced Technology of Materials, Ministry of Education.

School of Materials Science and Engineering, Southwest Jiaotong University, Chengdu, Sichuan, 610031, China

[b] Medical College of Acu-Moxi and Rehabilitation, Guanzhou University of Chinese Medicine

Guangzhou, Guangdong, 510006, China

1. Supplementary figure

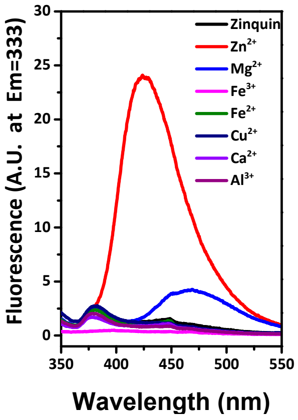

Figure S1. Specific fluorescence detection of Zinquin measured by fluorescence spectrophotometer.

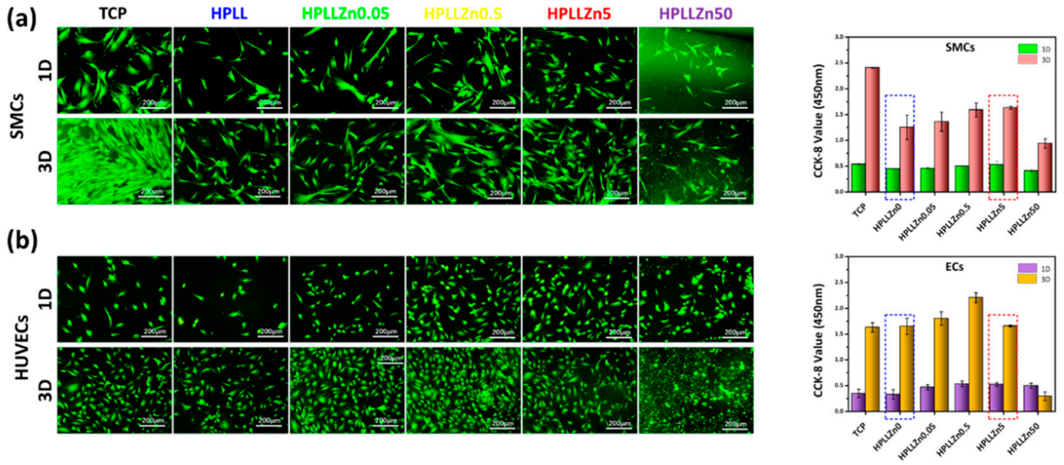

Figure S2. The influence of different zinc ion concentration gradient on cell viability. The cell viabilities were detected after the incubation with ECs (A) and SMCs (B) for 1 day and 3 days, separately. The highest zinc ion concentration that can maintain cell viability was selected for the research object. (HPLLZn0 was renamed HPLL, and HPLLZn5 was renamed HPLLZn in the full paper).

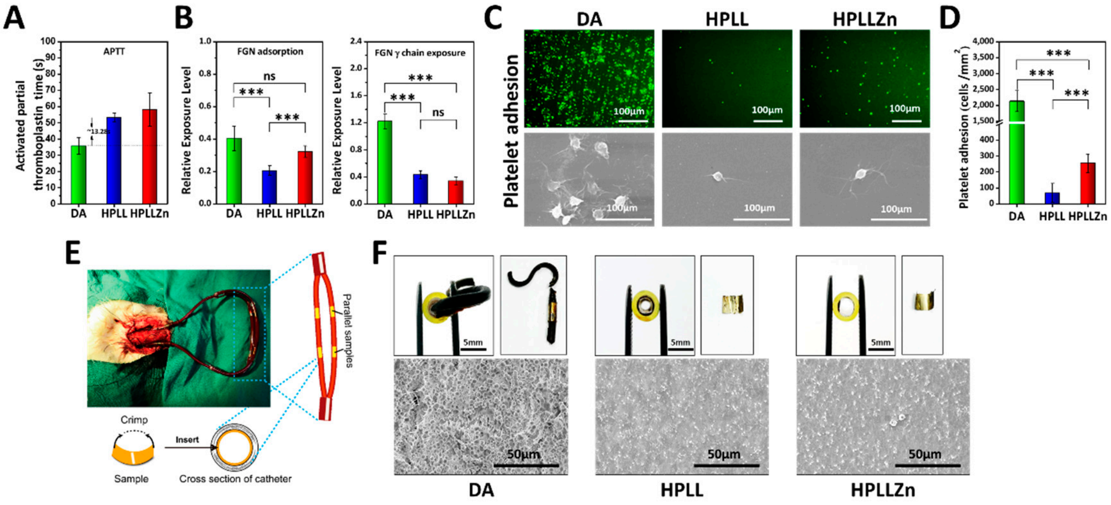

Figure S3. Evaluation of the performance of anticoagulation. (A) Activated partial thromboplastin time test. (B) Fibrinogen adsorption and the fibrinogen γ chain exposure. (C) Rhodamine 123 fluorescence images and SEM images of the platelet adhesion. (D) Quantitative statistics of the platelet adhesion. (E) The schematic of ex-vivo blood

circulation through the New Zealand white rabbit's carotid arteriovenous and the samples preparation. (F) Occlusion results of different coatings after 2-hours ex-vivo blood circulation and the thrombus formation on different coatings.

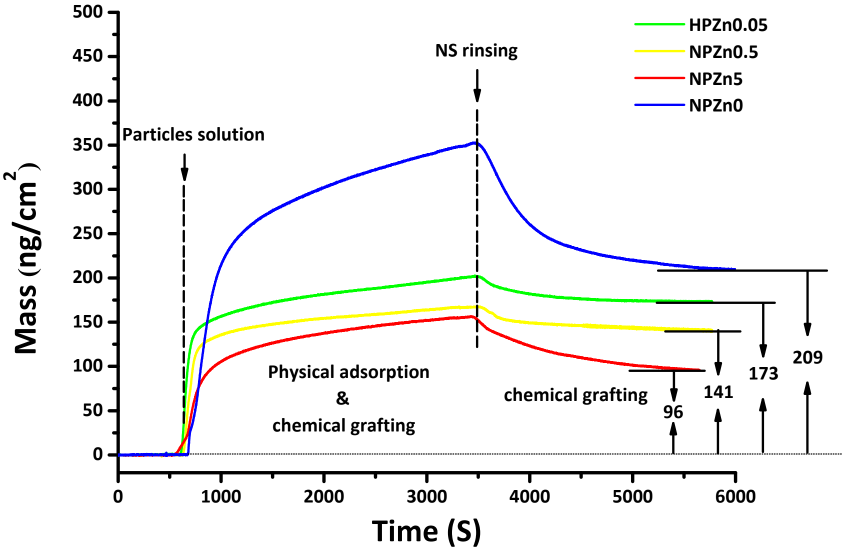

Figure S4. Real-time observation of complexes immobilization by quartz crystal microbalance with dissipation (QCM-D)

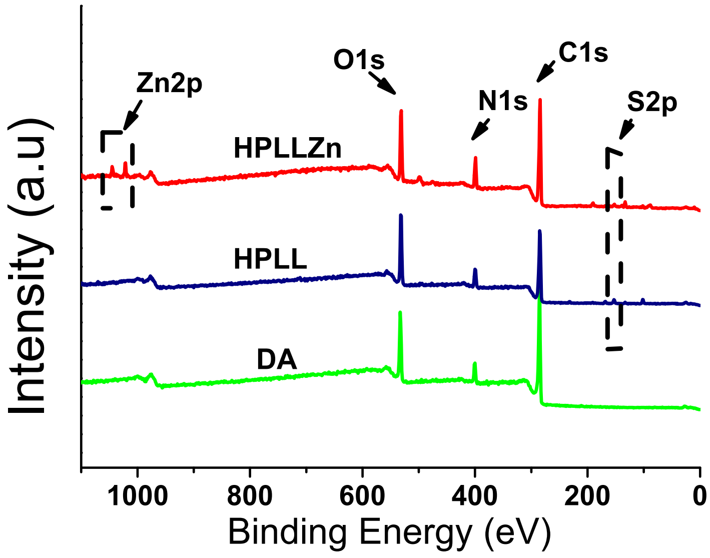

Figure S5. XPS wide-scan of HPLL particles and HPLLZn particles modified coatings

2. Supplementary experiment

S2.1 APTT assays

Activated partial thromboplastin time (APTT) were measured to evaluate the influence on the coagulation enzyme system. In detail, 500μl PPP added to the samples and incubated at 37°C for 30 min. After that, 100μl incubated PPP was transferred to the test tube, followed by addition of 100μl APTT agent and incubated at 37°C for 3min.Subsequently 100μl 0.025M $CaCl_2$ was added and the clotting time was measured in an automatic blood coagulation analyzer (ACL-200, Beckman Coulter, USA). Data were presented as mean ± SD (n = 3)

S2.2. Fibrinogen adsorption and the degeneration

Fibrinogen is the risk protein factors to effect thrombus form. The experiments to investigated fibrinogen adsorption and degeneration were very necessary and the operation was to add the platelet-poor plasma (PPP) on the sample surface incubated at 37°C. After 2h later, rinsed three times with PBS and blocked the nonspecific adsorption by bull serum albumin (1:10 in PBS) at 37°C for 1h. Subsequently the first antibody (mouse anti-human fibrinogen antibody (1:1000 in PBS) for adsorption and mouse monoclonal to anti-human fibrinogen gamma chain antibody (ab136520) for degeneration) were added on the sample surface and incubated at 37°C for 1h. Washed three times with PBS for 3min each and added 100μl Horseradish peroxidase (HRP)-conjugated goat anti-mouse IgG antibody (second antibody, 1:5000 in PBS) on the surface incubated at 37°C for 30 min. After that rinsed three times with PBS for 3min each, 100μl TMB agent was added to the sample surface and reacted for 10min. The color reaction was stopped by adding 50μl of 1M H_2SO_4 and 100μl of the reaction

solution was transferred to a 96-well plate; the absorbance was measured at 450nm. Data were presented as mean ± SD (n = 4)

S2.3. Platelet adhesion

Platelets-rich plasma (PRP) were obtained from fresh human whole blood by centrifuged at 1500rpm for 15min. Then the samples were immersed into the 0.5mL PRP and incubated at 37°C for 30min. Subsequently, the sample were rinsed three times with PBS for seconds. Fixed in 2.5% glutaraldehyde for 12h at RT. Finally, the samples stained by Rhodamine 123 fluorescent dye. Data were presented as mean ± SD (at least 8 pictures for cell counting)

S2.4. Ex-vivo blood circulation assessment of antithrombus

DA coating was prepared on the 316SS foils and immersed in different particles coatings solution at 20 °C with shock on table concentrator for 12-hours. The foils with different coating insert to the catheter which go through the New Zealand white rabbit' s carotid arteriovenous. After 2-hours blood flow, the catheters were collected and fixed with 4% paraformaldehyde overnight. (n = 2)

S2.5. Cell lines and culture conditions

ECs were isolated from human umbilical vein and SMCs were isolated from human umbilical artery. The details are available from the previous work[1].

[1] T. Liu, Y. Liu, Y. Chen, S. Liu, M.F. Maitz, X. Wang, K. Zhang, J. Wang, Y. Wang, J. Chen, N. Huang, Immobilization of heparin/poly-(L)-lysine nanoparticles on dopamine-coated surface to create a heparin density gradient for selective direction of platelet and vascular cells behavior, Acta Biomater. 10(5) (2014) 1940-1954.
